# Effect of Electrical Stimulation of the Quadriceps Muscles in the Management of Haemophilic Knee Arthropathy

**DOI:** 10.7759/cureus.105697

**Published:** 2026-03-23

**Authors:** Monia Hafiz, Mohammad Moniruzzaman, Mohd. Aziz Khan, Shohel Ahmed, Md. Jouel Ahamed, Shahina Sarker, Md. Sakib-Al-Nahian

**Affiliations:** 1 Physical Medicine and Rehabilitation, Farazy Hospital Ltd, Dhaka, BGD; 2 Physical Medicine and Rehabilitation, Dhaka Medical College, Dhaka, BGD; 3 Nutrition, United Nations Children's Fund (UNICEF) Bangladesh, Dhaka, BGD; 4 Physical Medicine and Rehabilitation, Ahsania Mission Cancer and General Hospital, Dhaka, BGD; 5 Physical Medicine and Rehabilitation, Chandpur Sadar Upazila Health Complex, Chandpur, BGD; 6 Physical Medicine and Rehabilitation, Savar Upazila Health Complex, Savar, BGD; 7 Physical Medicine and Rehabilitation, Green Life Medical College, Dhaka, BGD

**Keywords:** electrical stimulation, hemophilia, hemophilic arthropathy, isometric exercise, knee, womac

## Abstract

Background

Hemarthrosis is the most common disabling manifestation of haemophilia, and recurrent knee bleeding leads to haemophilic arthropathy with pain, muscle wasting, and functional limitation. Evidence on the added benefit of electrical stimulation (ES) in conjunction with therapeutic exercise for haemophilic knee arthropathy is limited, particularly in low-resource settings. So, this study was planned to evaluate the effect of quadriceps ES as an adjunct to isometric quadriceps exercise (IQE) on pain, muscle strength, muscle bulk, bleeding events, and functional status in patients with haemophilic knee arthropathy.

Methods

This quasi-experimental study was conducted at Dhaka Medical College Hospital from October 2021 to September 2022. Patients aged 12-41 years with mild-moderate haemophilia and mild-moderate haemophilic knee arthropathy were assigned purposively to Group A (ES + IQE; n = 25) or Group B (IQE alone; n = 26). IQE was prescribed twice daily for six months. Group A additionally received quadriceps ES (50 Hz, 15 minutes) twice weekly for the first three months. Outcomes (pain by visual analogue scale (VAS), muscle power by Medical Research Council (MRC) scale, muscle bulk by mid-thigh circumference, bleeding events, and functional outcome by Bangla validated Western Ontario and McMaster Osteoarthritis (WOMAC)) were assessed monthly for six months.

Results

Baseline characteristics were comparable between the groups. From the third follow-up onward, Group A showed significantly lower pain scores compared with Group B (VAS: 4.08 ± 0.64 vs 4.87 ± 0.86; p-value: 0.002), which remained significant at the sixth follow-up (4.00 ± 0.76 vs 4.69 ± 0.92; p-value: 0.006). Muscle strength improved significantly in Group A, with 52.0% achieving MRC grade 5 compared with 15.4% in Group B at the third follow-up (p-value: 0.006). Muscle bulk was also significantly greater in Group A from the third follow-up (34.89 ± 4.35 cm vs 31.42 ± 3.48 cm; p-value: 0.011). Functional outcomes improved significantly in the intervention group, with lower WOMAC total scores from the second follow-up (38.84 ± 5.15 vs 44.62 ± 4.67; p-value: 0.008) and highly significant differences by the sixth follow-up (p-value: <0.001). Bleeding events were infrequent and did not differ significantly between the groups during the follow-up period.

Conclusions

Quadriceps ES combined with IQE provides superior pain relief, strength gains, muscle bulk, and functional improvement, without increasing bleeding risk, in mild-to-moderate haemophilic knee arthropathy.

## Introduction

Haemophilia is an X-linked genetic bleeding disorder caused by a total or partial lack of coagulation factors -- factor VIII in haemophilia A (classic haemophilia) and factor IX in haemophilia B (Christmas disease). The estimated prevalence of haemophilia A is one in every 5,000 male live births and one in every 30,000 for haemophilia B [1,2]. According to the World Federation of Hemophilia's (WFH) Annual Global Survey 2017, Bangladesh has 1,249 registered haemophilia patients, 1,044 with haemophilia A and 199 with haemophilia B [3].

Hemarthrosis is the most common and clinically debilitating manifestation of haemophilia, with the knee joint accounting for nearly half of all bleeding episodes [4]. Recurrent intra-articular haemorrhage initiates a cascade of pathological events, including chronic synovial inflammation, progressive cartilage degeneration, and eventual development of haemophilic arthropathy [5].

This progressive joint pathology results in persistent pain, joint instability, deformity, muscle atrophy, restricted range of motion (ROM), and substantial functional limitation, ultimately compromising quality of life [6,7]. Chronic pain associated with haemophilic arthropathy commonly leads to a 20-30% reduction in muscle strength, largely attributable to pain-induced avoidance behaviours and kinesiophobia. Such disuse further accelerates muscle wasting, disrupts neuromuscular coordination, and perpetuates a vicious cycle of functional decline and progressive joint deterioration [8].

The gold standard for haemophilia management is prophylactic replacement therapy via periodic intravenous infusion of the deficient clotting factor [9]. Upon the onset of degenerative joint alterations, therapy approaches predominantly include physiotherapeutic and orthopaedic procedures designed to enhance functional ability and quality of life [10]. Exercise is an essential element of physiotherapy, and progressive strengthening combined with balance training has demonstrated safety and efficacy in persons with haemophilic arthropathy [11,12]. As arthropathy advances, physiotherapy programs may be enhanced by integrating manual therapy techniques, electrical stimulation (ES), and customised exercise modalities [13]. Manual treatment has been shown to be useful in enhancing joint range of motion (ROM) and alleviating discomfort in individuals with lower-limb haemophilic arthropathy [14-16]. Moreover, electrical stimulation has demonstrated a substantial improvement in muscle strength among individuals with haemophilia [17].

However, there is a notable lack of well-designed studies evaluating the additive or synergistic effects of electrical stimulation when combined with conventional exercise therapy on pain reduction, muscle strength, and functional outcomes in individuals with haemophilic knee arthropathy. Although physiotherapy is widely recognised as a cornerstone of management, evidence supporting the combined use of electrical stimulation and therapeutic exercise remains limited, particularly in low-resource settings. Conducting this study in Bangladesh is especially relevant given the high burden of haemophilia, limited access to regular factor replacement therapy, and restricted availability of specialised rehabilitation services. Many patients develop haemophilic arthropathy as a consequence of recurrent joint bleeding and inadequate long-term management. In this context, evaluating the effectiveness of electrical stimulation as an adjunct to conventional physiotherapy may provide valuable insights into cost-effective and feasible rehabilitation strategies. Therefore, this study aimed to evaluate the effect of electrical stimulation of the quadriceps muscles on pain, muscle strength, functional status, and clinical outcomes in patients with haemophilic knee arthropathy.

## Materials and methods

A quasi-experimental study was conducted between October 2021 and September 2022 among patients with haemophilia A and B presenting with knee arthropathy. Participants were referred from the Department of Haematology and the Bone Marrow Transplant Unit to the Department of Physical Medicine and Rehabilitation, Dhaka Medical College Hospital (DMCH), Dhaka, Bangladesh. The study was conducted in accordance with the principles of the Declaration of Helsinki for research involving human participants and received ethical approval from the Dhaka Medical College Ethical Review Committee (ERC) (Memo No. ERC-DMC/ECC/2021/273).

Selection of the study subjects

This study included patients aged 12-41 years with mild and moderate haemophilia and mild and moderate haemophilic knee arthropathy. Patients with severe haemophilia and haemophilic knee arthropathy, active joint bleeding or infection, and living outside Dhaka were excluded from the study. Mild and moderate haemophilia was defined according to the recommendations of the scientific subcommittee on factor VIII and factor IX of the scientific and standardisation committee of the International Society on Thrombosis and Haemostasis [18]. Mild and moderate haemophilic knee arthropathy was determined according to the Arnold-Hilgartner classification of haemophilic knee arthropathy, where stages I and II are considered mild haemophilic knee arthropathy, and stages Ⅲ and Ⅳ are considered moderate haemophilic knee arthropathy [19].

The aims and objectives of the study were discussed with the patients, and informed written consent/assent was obtained. Initially, 58 patients fulfilled the selection criteria and were approached for the study, but eventually only 51 patients were included. At baseline, participant demographic data (age, body mass index (BMI), etc.), radiological grading of haemophilic knee arthropathy, pain status, bulk of muscle, muscle power, bleeding events, and functional status were recorded.

Allocation of treatment/intervention

Participants were assigned purposively to the intervention group (Group A) or the control group (Group B). Among the participants, 25 patients were allocated to Group A for electrical stimulation (ES) along with isometric quadriceps exercise (IQE) and 26 patients to Group B for IQE alone.

Interventions/treatment

Both patients of Group A and Group B received 10 repetitions/session of IQE two times daily for six months. Isometric quadriceps exercise was given in the form of extension of the knees with the hip at neutral. Patients were placed in a supine position. A rolled-up towel was placed beneath the knee. They were instructed to push their knee back to maximally activate their thigh muscles to straighten their knee and dorsiflex the ankle, and hold the contraction for 10 seconds and relaxation for five seconds (10 repetitions/session) [20]. For the home exercise program, the patients were given an exercise schedule form at each follow-up and requested to mark each time after completion of IQE as instructed with a (√) mark. Then these were collected from them at each follow-up. In between physical follow-ups, they were also contacted over mobile phone weekly.

For Group A patients, in addition to IQE, patients were treated with ES of the quadriceps muscle for two times/week (Saturday and Tuesday) for the initial three months. For ES, bipolar electrodes and an electrostimulator apparatus were used for ES (manufactured locally). The cathode (blue) was placed on the quadriceps muscle at the height of the greater femoral trochanter, and the anode (red) was placed 3 cm superior to the patella and medial to the midline of the patella [21]. The frequency used in each session was 50 Hz with intensity according to patient compliance for 15 minutes with isometric quadriceps exercise.

Outcome assessment

**Figure 1:**
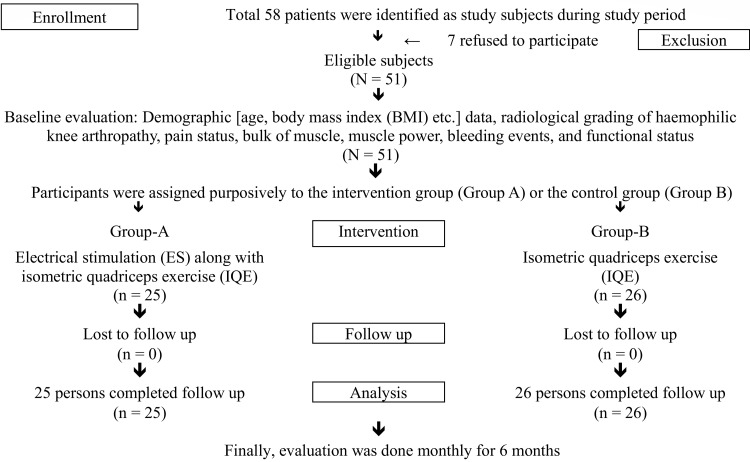
Study flow chart.

Patients were followed up monthly for six months. At each follow-up, the patient was evaluated for pain, muscle strength, bulk of muscle, bleeding events, and functional status. A visual analogue scale was used for assessment of pain, and the Bangla validated Western Ontario and McMaster Osteoarthritis (WOMAC) was used to assess the functional status of the knee joint [22]. For the measurement of the bulk of the quadriceps muscles, a measuring tape was used. The bulk of the muscle was measured around mid-thigh circumference (in cm). Muscle power was measured using the Medical Research Council (MRC) Muscle Power Scale, which grades muscle power on a scale of 0 to 5 relative to the maximum expected for that muscle. where 0 = no contraction, 1 = flicker or trace of contraction, 2 = active movement, with gravity eliminated, 3 = active movement against gravity, 4 = active movement against gravity and resistance, and 5 = normal power [23]. A flow diagram of the subject recruitment process for the study is provided in Figure 1.

Statistical analysis

Data were analysed using the Statistical Package for the Social Sciences (SPSS) software (version 23) (IBM Corp., Armonk, New York, USA). Continuous variables were expressed as mean ± standard deviation (SD), while categorical variables were presented as frequency and percentage. Normality of continuous data was assessed before analysis. Comparisons of continuous variables between Group A and Group B at baseline and at different follow-up periods were performed using the independent sample t-test. Categorical variables were compared using the Chi-square test or Fisher’s exact test, as appropriate, based on expected cell counts. Muscle strength assessed by Medical Research Council (MRC) grading was analysed as a categorical variable. All statistical tests were two-tailed, and a p-value of <0.05 was considered statistically significant. The results were presented in tabular and graphical formats where appropriate.

## Results

The age distribution showed that the majority of participants in Group A were within the 12-20-year age group (15 (60.0%)), whereas most participants in Group B belonged to the 21-30-year age group (14 (53.8%)); however, this difference was not statistically significant (p-value: 0.084). The mean ages of Group A (21.0 ± 6.4 years) and Group B (21.8 ± 7.1 years) also did not differ significantly (p-value: 0.685). Regarding body mass index, the majority of participants in both groups had normal BMI values (18 (72.0%) in Group A and 19 (73.1%) in Group B). The proportions of underweight and overweight participants were similar between the groups, and no statistically significant difference in BMI distribution was observed (p-value: 0.999). Overall, the two groups were comparable with respect to baseline demographic characteristics, suggesting appropriate group allocation before intervention (Table 1).

**Table 1:** Demographic characteristics of participants in Group A and Group B. Group A: Electrical stimulation (ES) along with isometric quadriceps exercise (IQE); Group B: Isometric quadriceps exercise (IQE). ᵃIndependent t-test, ^b^Fisher's exact test was done. Data presented as frequency (percentage) and mean ± SD.

Demographic characteristics		Group A (n = 25)	Group B (n = 26)	p-value
Age (years)	12-20	15 (60.0)	11 (42.3)	0.084^b^
	21-30	6 (24.0)	14 (53.8)	
	31-41	4 (16.0)	1 (3.8)	
	Mean ± SD	21.0 ± 6.4	21.8 ± 7.1	0.685ᵃ
Body mass index	Underweight	4 (16.0)	5 (19.2)	0.999^b^
	Normal	18 (72.0)	19 (73.1)	
	Overweight	3 (12.0)	2 (7.7)	

**Figure 2:**
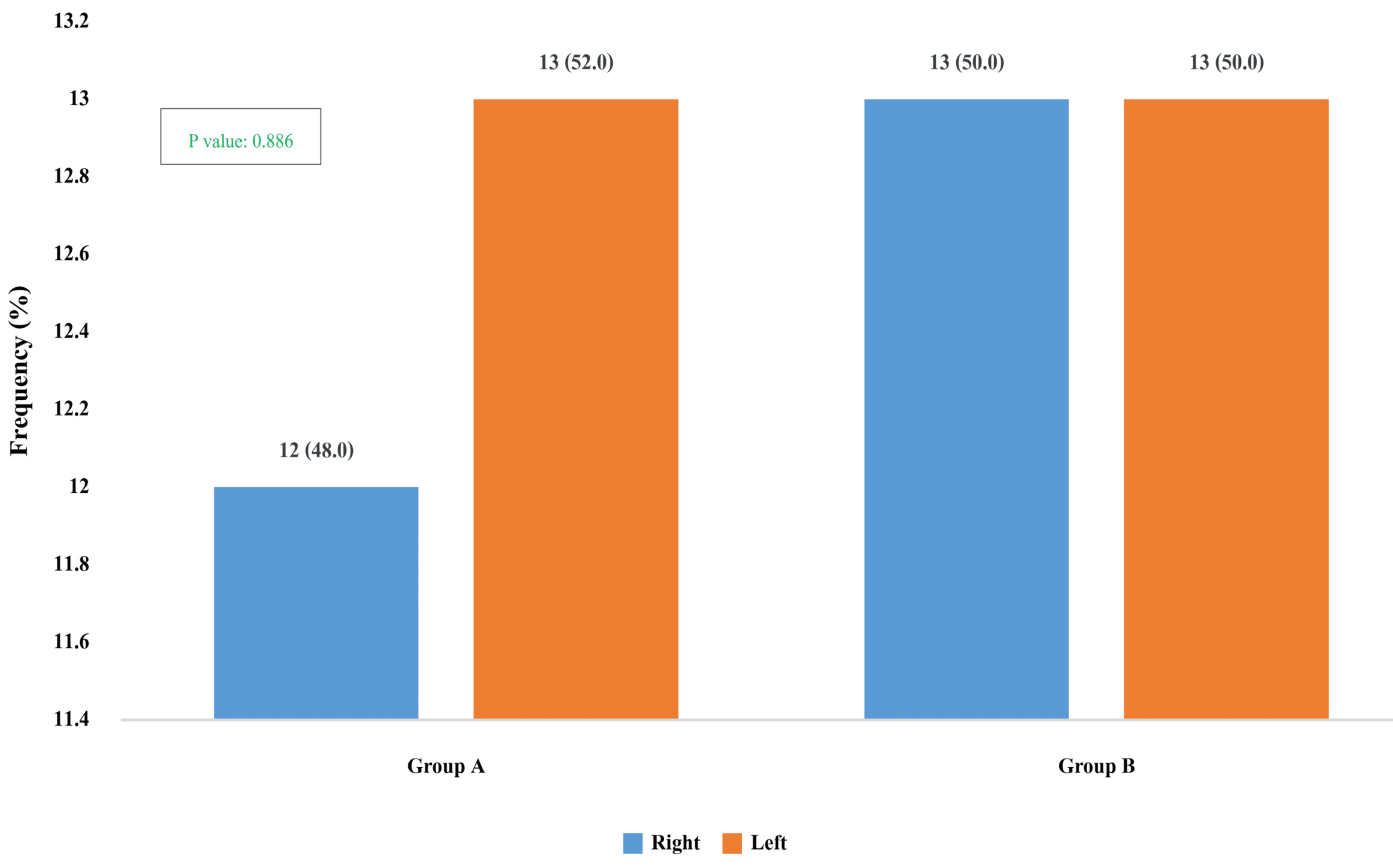
Distribution of participants according to the side of knee involvement in Group A and Group B. Group A: Electrical stimulation (ES) along with isometric quadriceps exercise (IQE); Group B: Isometric quadriceps exercise (IQE).

In Group A, the left knee was more frequently involved (13 participants, 52.0%) than the right (12 participants, 48.0%). In contrast, Group B showed an equal distribution of knee involvement, with 13 participants (50.0%) having right-sided and 13 participants (50.0%) having left-sided knee arthropathy. There was no statistically significant difference in the distribution of the affected side between the two groups (p-value: 0.886) (Figure 2).

**Figure 3:**
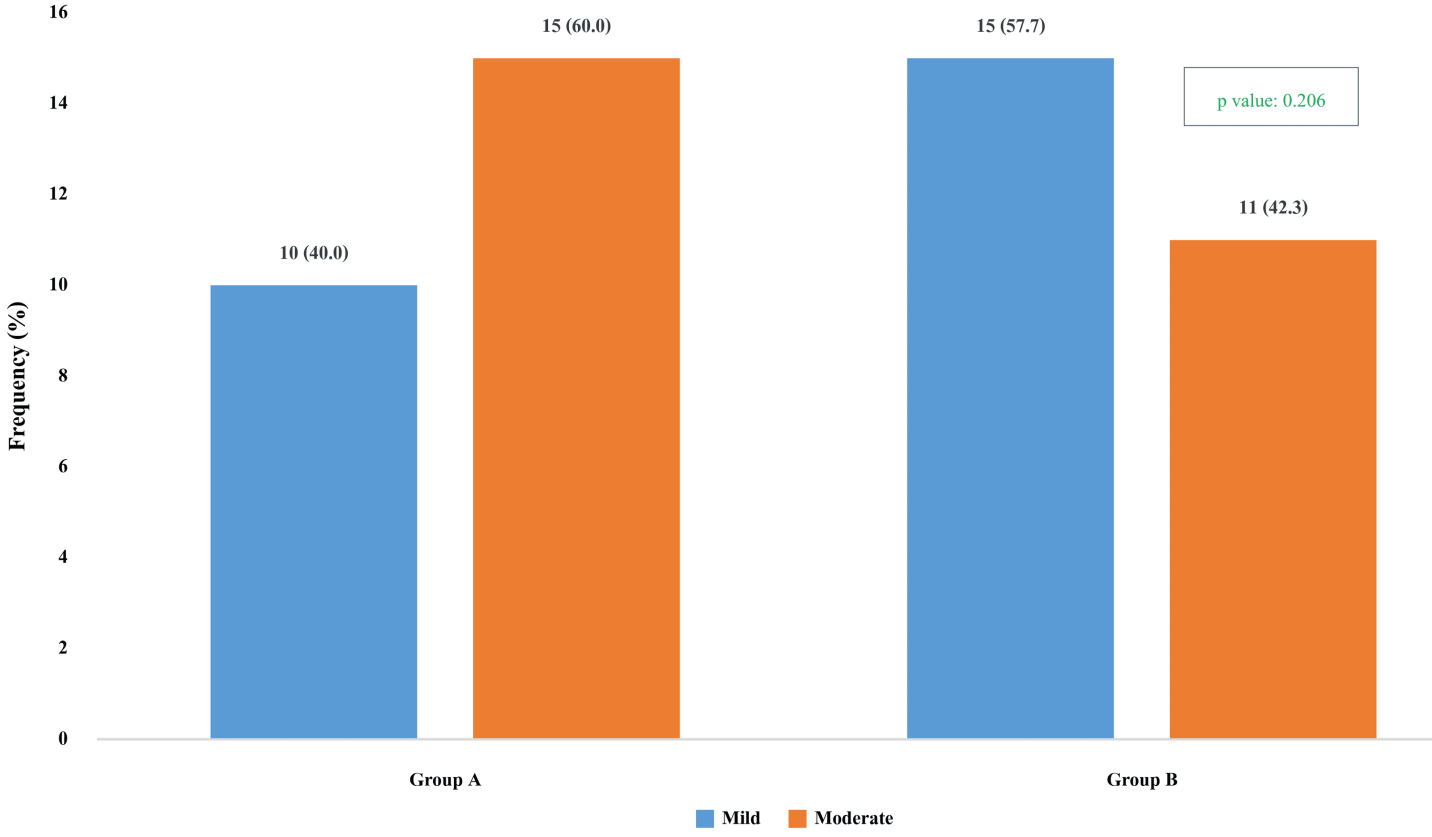
Distribution of the participants according to the stages of knee arthropathy in Group A and Group B. Group A: Electrical stimulation (ES) along with isometric quadriceps exercise (IQE); Group B: Isometric quadriceps exercise (IQE).

In Group A, the majority of participants presented with moderate knee arthropathy [15 (60.0%)], while 10 (40.0%) had mild disease. Conversely, in Group B, mild knee arthropathy was more frequent, observed in 15 participants (57.7%), whereas 11 participants (42.3%) had moderate involvement. Although a higher proportion of moderate knee arthropathy was observed in Group A and mild knee arthropathy in Group B, the difference between the groups was not statistically significant (p-value: 0.206) (Figure 3).

Pain scores were comparable between the groups at baseline (6.04 ± 0.67 vs 5.73 ± 0.45; p-value: 0.060) and during the first two follow-ups (p-value: >0.05). From the third follow-up onward, Group A showed significantly lower pain scores than Group B, including at the third follow-up (4.08 ± 0.64 vs 4.87 ± 0.86; p-value: 0.002) and sixth follow-up (4.00 ± 0.76 vs 4.69 ± 0.92; p-value: 0.006). Muscle strength assessed by MRC grading was similar initially but improved significantly in Group A from the third follow-up, where 52.0% achieved grade 5 compared with 15.4% in Group B (p-value: 0.006). Muscle bulk also became significantly greater in Group A from the third follow-up (34.89 ± 4.35 cm vs 31.42 ± 3.48 cm; p-value: 0.011) and remained higher at the sixth follow-up (34.87 ± 4.30 cm vs 31.44 ± 3.37 cm; p-value: 0.007). Bleeding events were infrequent and comparable between the groups throughout follow-up (16.0% vs 11.5% at the first follow-up; p-value: 0.703). Overall, pain, muscle strength, and muscle bulk were comparable at baseline but improved significantly in Group A from the third follow-up onward compared with Group B, while bleeding events remained infrequent and similar between the groups (Table 2).

**Table 2:** Comparison of clinical and functional outcomes between Group A and Group B during baseline and follow-ups. Group A: Electrical stimulation (ES) along with isometric quadriceps exercise (IQE); Group B: Isometric quadriceps exercise (IQE). ^a^Independent t-test, ^b^Chi-square test, and ^c^Fisher's exact test was done. Data presented as frequency (percentage), mean ± SD.

Variables			Group A (n = 25)	Group B (n = 26)	p-value
Pain	Baseline		6.04 ± 0.67	5.73 ± 0.45	0.060ᵃ
	At first follow-up		5.36 ± 0.81	5.15 ± 0.67	0.328ᵃ
	At second follow-up		4.40 ± 0.71	4.95 ± 0.88	0.062ᵃ
	At third follow-up		4.08 ± 0.64	4.87 ± 0.86	0.002ᵃ
	At fourth follow-up		4.04 ± 0.68	4.75 ± 0.87	0.003ᵃ
	At fifth follow-up		4.00 ± 0.73	4.73 ± 0.87	0.003ᵃ
	At sixth follow-up		4.00 ± 0.76	4.69 ± 0.92	0.006ᵃ
Medical Research Council (MRC) grading	Baseline	2–4	23 (92.0)	23 (88.5)	0.999^c^
		5	2 (8.0)	3 (11.5)	
	At first follow-up	2–4	21 (84.0)	23 (88.5)	0.703^c^
		5	4 (16.0)	3 (11.5)	
	At second follow-up	2–4	18 (72.0)	23 (88.5)	0.173^c^
		5	7 (28.0)	3 (11.5)	
	At third follow-up	2–4	12 (48.0)	22 (84.6)	0.006^b^
		5	13 (52.0)	4 (15.4)	
	At fourth follow-up	2–4	12 (48.0)	22 (84.6)	0.006^b^
		5	13 (52.0)	4 (15.4)	
	At fifth follow-up	2–4	11 (44.0)	22 (84.6)	0.002^b^
		5	14 (56.0)	4 (15.4)	
	At sixth follow-up	2–4	11 (44.0)	22 (84.6)	0.002^b^
		5	14 (56.0)	4 (15.4)	
Bulk of muscle (cm)	Baseline		33.0 ± 4.64	31.30 ± 3.53	0.148^a^
	At first follow-up		33.42 ± 4.77	31.35 ± 3.66	0.105^a^
	At second follow-up		33.83 ± 4.56	31.40 ± 3.62	0.063^a^
	At third follow-up		34.89 ± 4.35	31.42 ± 3.48	0.011^a^
	At fourth follow-up		34.88 ± 4.37	31.43 ± 3.39	0.009^a^
	At fifth follow-up		34.88 ± 4.30	31.44 ± 3.36	0.007^a^
	At sixth follow-up		34.87 ± 4.30	31.44 ± 3.37	0.007^a^
Bleeding events	At first follow-up		4 (16.0)	3 (11.5)	0.703^c^
	At second follow-up		3 (12.0)	2 (7.7)	0.668^c^
	At third follow-up		2 (8.0)	2 (8.0)	0.999^c^
	At fourth follow-up		1 (4.0)	2 (7.7)	0.999^c^
	At fifth follow-up		0 (0.0)	1 (3.8)	0.999^c^
	At sixth follow-up		0 (0.0)	1 (3.8)	0.999^c^

Baseline WOMAC pain, stiffness, physical function, and total scores were comparable between Group A and Group B (p-value: >0.05). From the second follow-up onward, Group A showed significantly greater improvement than Group B across all WOMAC domains. Pain scores were significantly lower in Group A from the second follow-up (8.38 ± 0.58 vs 9.62 ± 1.41; p-value: 0.042) and remained highly significant through the sixth follow-up (p-value: <0.001). Similar patterns were observed for stiffness and physical function, with significant differences emerging from the second follow-up (p-value: 0.046) and persisting thereafter. Consequently, total WOMAC scores were significantly lower in Group A from the second follow-up onward (38.84 ± 5.15 vs 44.62 ± 4.67; p-value: 0.008), indicating superior overall clinical improvement with electrical stimulation combined with isometric quadriceps exercise compared with isometric exercise alone (Table 3).

**Table 3:** Comparison of Western Ontario and McMaster Universities Osteoarthritis (WOMAC) Index scores between Group A and Group B at different follow-up periods. Group A: Electrical stimulation (ES) along with isometric quadriceps exercise (IQE); Group B: Isometric quadriceps exercise (IQE). Independent t-test was done. Data presented as mean ± SD.

Variables		Group A (n = 25)	Group B (n = 26)	p-value
Pain	Baseline	11.4 ± 1.1	11.0 ± 1.0	0.142
	At first follow-up	9.23 ± 1.33	10.53 ± 1.50	0.068
	At second follow-up	8.38 ± 0.58	9.62 ± 1.41	0.042
	At third follow-up	7.92 ± 1.72	9.30 ± 1.74	0.002
	At fourth follow-up	5.36 ± 0.99	9.26 ± 1.76	<0.001
	At fifth follow-up	5.36 ± 0.99	9.23 ± 1.73	<0.001
	At sixth follow-up	5.36 ± 1.22	9.23 ± 1.73	<0.001
Stiffness	Baseline	4.2 ± 1.0	3.7 ± 0.8	0.060
	At first follow-up	3.54 ± 1.09	3.62 ± 0.8	0.051
	At second follow-up	2.88 ± 1.03	3.48 ± 0.76	0.023
	At third follow-up	2.16 ± 1.03	2.92 ± 0.84	0.005
	At fourth follow-up	1.54 ± 0.89	2.07 ± 0.71	0.002
	At fifth follow-up	1.24 ± 0.89	1.95 ± 0.68	0.002
	At sixth follow-up	1.25 ± 0.98	1.90 ± 0.71	0.002
Physical function	Baseline	36.16 ± 4.14	35.07 ± 2.50	0.261
	At first follow-up	32.44 ± 4.35	33.88 ± 2.63	0.659
	At second follow-up	27.24 ± 4.37	31.12 ± 3.36	0.046
	At third follow-up	21.00 ± 3.17	29.84 ± 4.47	0.008
	At fourth follow-up	20.08 ± 3.18	28.19 ± 4.55	<0.001
	At fifth follow-up	19.76 ± 3.33	27.35 ± 4.27	<0.001
	At sixth follow-up	19.68 ± 3.37	26.04 ± 4.28	<0.001
WOMAC total	Baseline	51.8 ± 4.6	49.8 ± 3.9	0.093
	At first follow-up	44.60 ± 5.3	47.53 ± 4.9	0.081
	At second follow-up	38.84 ± 5.15	44.62 ± 4.67	0.008
	At third follow-up	30.08 ± 4.46	40.92 ± 6.64	0.001
	At fourth follow-up	26.48 ± 4.09	38.62 ± 6.34	<0.001
	At fifth follow-up	26.16 ± 4.27	35.23 ± 5.90	<0.001
	At sixth follow-up	26.06 ± 4.38	34.92 ± 5.84	<0.001

## Discussion

The present study evaluated the effectiveness of electrical stimulation (ES) combined with isometric quadriceps exercise (IQE) compared with IQE alone in patients with haemophilic knee arthropathy. The findings demonstrate that the addition of electrical stimulation resulted in significantly greater improvements in pain reduction, muscle strength, muscle bulk, and functional outcomes over a six-month follow-up period. These results support the role of adjunctive electrical stimulation as an effective rehabilitation strategy in patients with haemophilic arthropathy, particularly in resource-limited settings.

**Figure 4:**
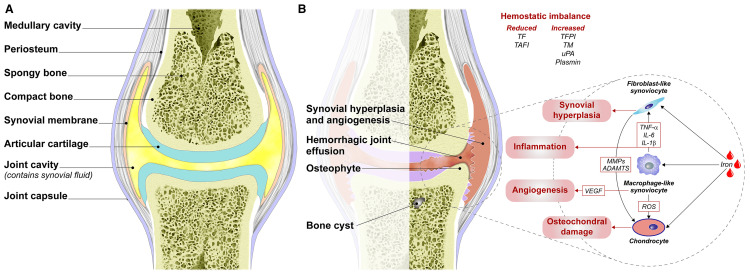
(A) Normal synovial joint structure and (B) pathological changes in haemophilic arthropathy. (A) Normal synovial joint structure showing smooth articular cartilage, synovial membrane, and synovial fluid that facilitate painless movement and proper joint lubrication. (B) Haemophilic joint changes are characterised by synovial hyperplasia, angiogenesis, recurrent hemarthrosis, osteophyte formation, and subchondral bone cysts, leading to inflammation, cartilage destruction, and progressive joint dysfunction. ADAMTS: a disintegrin and metalloproteinase with thrombospondin motifs; IL‐1: interleukin‐1; IL‐6: interleukin‐6; MMPs: metalloproteinases; ROS: reactive oxygen species; TAFI: thrombin activatable fibrinolysis inhibitor; TF: tissue factor; TFPI: tissue factor pathway inhibitor; TM: thrombomodulin; TNF‐alpha: tumor necrosis factor‐alpha; uPA: urokinase plasminogen activator; VEGF: vascular endothelial growth factor. Source: Figure has been adapted from an open-source journal, Gualtierotti et al. (2021) [24], licensed under a Creative Commons Attribution-NonCommercial-NoDerivatives 4.0 International (CC BY-NC-ND 4.0) (https://creativecommons.org/licenses/by/4.0/).

Recurrent intra-articular haemorrhage leads to chronic synovial inflammation, cartilage degeneration, and progressive haemophilic arthropathy (Figure 4) [24], resulting in pain, joint deformity, restricted motion, and reduced quality of life [6,7]. At baseline, pain, reduced muscle strength, and impaired functional status were evident among the study participants. At baseline, both groups were comparable in terms of age, body mass index, severity of arthropathy, pain scores, muscle strength, and functional status, indicating the minimisation of the risk of selection bias. The absence of significant baseline differences strengthens the validity of the observed treatment effects and suggests that subsequent improvements can be attributed primarily to the intervention rather than pre-existing disparities.

Several studies have demonstrated that exercise plays a significant role in reducing pain among individuals with haemophilic arthropathy [14,15]. In the present study, both treatment groups exhibited a gradual improvement in pain over time; however, patients who received electrical stimulation experienced significantly greater pain relief from the third follow-up onward. Chronic pain in haemophilic arthropathy is primarily attributed to recurrent hemarthrosis, persistent synovial inflammation, and progressive joint degeneration [7]. Electrical stimulation may alleviate pain through multiple mechanisms, including enhanced neuromuscular activation, improved local blood circulation, and modulation of nociceptive transmission via the gate control theory [25,26]. The findings of this study are consistent with previous reports demonstrating reduced pain intensity following neuromuscular electrical stimulation in individuals with haemophilia [27]. The earlier onset and sustained nature of pain reduction observed in the intervention group suggest that electrical stimulation may augment the therapeutic effects of exercise alone, offering a more effective approach to pain management in haemophilic arthropathy.

Muscle strength improvement, as assessed by the Medical Research Council (MRC) grading, was significantly greater in the electrical stimulation group from the third follow-up onward. Querol et al. (2006) first reported that the application of electrical stimulation in haemophilic patients contributes to the gain and development of strength and trophism [28], and our study findings align with their findings. The improvement of muscle strength is evident in haemophilic arthropathy [14,16]. In this study, while both groups initially demonstrated comparable muscle strength, patients receiving combined therapy showed a progressive shift toward higher MRC grades over time. This finding supports the hypothesis that electrical stimulation enhances voluntary muscle activation [29] and counteracts disuse-related muscle atrophy [30] commonly seen in chronic joint pathology. In haemophilic arthropathy, recurrent pain and fear of bleeding often result in protective underuse [16], leading to muscle atrophy and reduced neuromuscular efficiency. Electrical stimulation likely mitigates these effects by promoting muscle fibre recruitment, improving neuromuscular coordination, and facilitating strength gains even in the presence of pain or limited voluntary effort [29,30].

Similarly, the increase in muscle bulk observed in the intervention group further reinforces the beneficial role of electrical stimulation, consistent with the findings reported by Gomis et al. (2009) [17]. Although early follow-up measurements did not show significant differences, a statistically significant increase in quadriceps circumference emerged from the third follow-up onward. This delayed but sustained hypertrophic response is consistent with the physiological timeline of muscle adaptation and supports the notion that neuromuscular electrical stimulation can augment muscle hypertrophy when combined with therapeutic exercise. In contrast, the control group showed minimal improvement in muscle bulk, highlighting the limitations of exercise alone in reversing chronic muscle wasting associated with haemophilic arthropathy.

Improvements in the functionality and quality of life during exercises are evident [14,16]. Functional outcomes, assessed using the WOMAC index, suggested such improvement in the exercise-alone group; however, marked superiority in functional outcome was demonstrated in the intervention group. Significant improvements in pain, stiffness, and physical function domains were evident from the second follow-up and continued throughout the study period. These findings indicate that the combined intervention not only improves isolated physical parameters but also translates into meaningful functional benefits in daily activities. Improvement in WOMAC scores reflects enhanced mobility, reduced joint stiffness, and better overall quality of life, which are key goals in the management of haemophilic arthropathy.

Several studies have reported that, with controlled physiotherapy/exercises, there was a limited frequency of bleeding episodes/hemarthrosis [14,27]. Importantly, the addition of electrical stimulation did not increase the frequency of bleeding episodes. Bleeding rates were low and comparable between groups throughout the study period, suggesting that electrical stimulation is a safe adjunctive modality when applied appropriately. The safety profile observed in this study supports the inclusion of electrical stimulation in comprehensive physiotherapy programs for haemophilic patients. However, literature specifically addressing electrical stimulation in haemophilic arthropathy remains limited, particularly in low- and middle-income countries. The present study contributes valuable evidence from a resource-constrained setting, demonstrating that a relatively low-cost and accessible intervention can significantly enhance functional recovery.

Limitations

Despite its strengths, this study has some limitations. The sample size was relatively small, and the follow-up period was limited to six months, which may not fully capture long-term disease progression or sustained benefits. Moreover, the inclusion of only patients with mild-to-moderate haemophilic arthropathy restricts the applicability of the findings to individuals with more advanced joint involvement. The absence of an objective assessment of the knee joint range of motion further limits the comprehensive evaluation of functional improvement. Future studies employing randomised allocation, larger and more heterogeneous cohorts, longer follow-up durations, and objective biomechanical or imaging outcome measures would provide stronger evidence regarding the effectiveness of combined therapeutic interventions in haemophilic arthropathy.

## Conclusions

This study demonstrates that the addition of electrical stimulation to conventional isometric quadriceps exercise offers significant clinical benefits in the management of haemophilic knee arthropathy. Patients receiving combined therapy experienced greater reductions in pain, improved muscle strength, increased muscle bulk, and superior functional outcomes compared to exercise alone. These improvements became evident from the mid-treatment period and were sustained throughout follow-up, without an associated increase in bleeding episodes. The findings suggest that electrical stimulation enhances neuromuscular activation and functional recovery beyond what can be achieved with exercise alone. Given its safety, feasibility, and effectiveness, electrical stimulation represents a valuable adjunct to physiotherapy in patients with haemophilic arthropathy, particularly in resource-limited settings. However, randomised controlled trials with extended follow-up are required to confirm these associations and to determine long-term effects on joint preservation and disease progression.
